# EEG frequency tagging to explore the cortical activity related to the tactile exploration of natural textures

**DOI:** 10.1038/srep20738

**Published:** 2016-02-08

**Authors:** Athanasia Moungou, Jean-Louis Thonnard, André Mouraux

**Affiliations:** 1 Institute of Neuroscience (IoNS), Université catholique de Louvain (UCL), Brussels, Belgium; 2 Cliniques Universitaires Saint-Luc, Physical and Rehabilitation Department, Université catholique de Louvain, Brussels, Belgium

## Abstract

When sliding our fingertip against a textured surface, complex vibrations are produced in the skin. It is increasingly recognised that the neural transduction and processing of these vibrations plays an important role in the dynamic tactile perception of textures. The aim of the present study was to develop a novel means to tag the cortical activity related to the processing of these vibrations, by periodically modulating the amplitude of texture exploration-induced vibrations such as to record a steady-state evoked potential (SS-EP). The EEG was recorded while the right index fingertip was scanned against four different textures using a constant exploration velocity. Amplitude modulation of the elicited vibrations was achieved by periodically modulating the force applied against the finger. Frequency analysis of the recorded EEG signals showed that modulation of the vibrations induced by the fingertip-texture interactions elicited an SS-EP at the frequency of modulation (3 Hz) as well as its second harmonic (6 Hz), maximal over parietal regions contralateral to the stimulated side. Textures generating stronger vibrations also generated SS-EPs of greater magnitude. Our results suggest that frequency tagging using SS-EPs can be used to isolate and explore the brain activity related to the tactile exploration of natural textures.

Perception of the external environment through touch is essentially a dynamic process involving movement such as repetitive stroking of a surface to explore its texture. In fact, when the fingertip is maintained static against a textured surface, identifying the texture is often difficult or even impossible. In contrast, when the fingertip is allowed to slide against the textured surface, it becomes possible to discriminate highly similar textures^1^,^2^,^3^.

Until recently, most studies in the field of touch perception have focused on the brain responses elicited by static stimuli such as static skin indentation, or the neural responses elicited by dynamic but artificial stimuli such as sinusoidal vibrations^4^, or very coarse textures such as gratings with a large and constant spatial period^3^,^5^,^6^ or Braille dot patterns^7^. To our knowledge, no studies have investigated the brain activity when stroking natural textures in humans.

At the level of peripheral mechanoreceptors, previous research focusing on very coarse textures, such as Braille dot patterns, suggested that the dynamic perception of textures is essentially reflected in the spatial pattern of activity elicited in slowly adapting Type I (SAI) mechanoreceptors, having very punctate receptive fields^8^. However, it is increasingly recognised that the dynamic perception of fine natural textures relies more on the transduction of high-frequency vibrations by rapidly-adapting (RA) and Pacinian (PC) mechanoreceptors^8^,^9^,^10^.

Therefore, the perception of coarse textures, such as gratings and Braille dot patterns, and the perception of fine natural textures, such as different kinds of cloth, probably involve different neural mechanisms^11^. The identification and discrimination of coarse textures would predominantly rely on a spatial decoding of the activity generated within populations of slowly-adapting SAI mechanoreceptors, whereas the identification of fine textures would predominantly rely on a temporal decoding of the frequency content of the activity generated within rapidly-adapting RA and PC mechanoreceptors^11^. This temporal mechanism implies that texture-elicited vibrations play an important role in texture perception^12^. Supporting this notion, it has been shown that ring anaesthesia of the index finger, by blocking the transmission of any input originating from slowly adapting mechanoreceptors of the index fingertip, has little or no effect on the ability of participants to discriminate different grains of sandpapers^13^. During anaesthesia, texture roughness discrimination would thus be achieved by the transduction and processing of high-frequency vibrations propagating in the index fingertip when scanning the texture^9^,^14^,^15^. Further supporting this hypothesis, Manfredi^16^ recorded the vibrations induced by exploring a wide range of textures encountered in daily life using a laser Doppler vibrometer, and showed that the different textures can be accurately classified based on the spectral content of the induced vibrations.

*How are textures represented at cortical level?* Single-unit recordings performed in animals have suggested that, at the level of the primary somatosensory cortex (SI), coarse textures are encoded spatially based on the differential activity generated within the population of neurons whose receptive fields map the activated skin surface^15^. In contrast, the cortical encoding of fine natural textures remains largely unknown. In his seminal study, Mountcastle *et al.* (1969) found that SI neurons are able to follow periodic input for frequencies up to 100–200 Hz^17^, suggesting that SI would be unable to achieve a temporal coding of the high-frequency vibrations typically generated by scanning natural textures. However, this notion has been challenged by recent findings showing that, although single units are unable to follow each cycle of very high-frequency vibrations, the firing of some single units can still exhibit some degree of phase locking for stimulation frequencies up to 800 Hz (4% out of 69 units, area 3b of SI)^18^. Therefore, when considered as a population, SI neurons may actually have the ability to achieve a temporal encoding of frequencies spanning the entire bandwidth of peripheral mechanoreceptors^18^,^19^.

Characterising, in humans, the cortical activity related to the perception of fine natural textures is technically challenging. Using scalp electroencephalography (EEG), studies have shown that mechanical sinusoidal vibration of the skin or repeated electrical activation of afferent fibres at a constant frequency can elicit a neuronal entrainment at cortical level, appearing as peaks in the EEG frequency spectrum, at frequencies corresponding to the frequency of stimulation and its harmonics^20^. When stimulating the hand, the scalp topography of these steady-state evoked potentials (SS-EPs) is maximal over the parietal region contralateral to the stimulated hand, suggesting that the elicited responses predominantly originate from SI. However, studies comparing the responses elicited by stimuli delivered at different frequencies have shown that reliable vibration-induced SS-EPs can only be obtained for stimulation frequencies below 50–100 Hz^21^,^22^,^23^. This could be related to the fact that EEG predominantly reflects slow post-synaptic activity rather than action potentials. Moreover, because of its capacitive properties, the scalp acts as a low pass filter attenuating high-frequency signals^24^.

Taking these limitations into consideration, the aim of the present study was to set the basis for a novel approach to capture the cortical processing of fine natural textures in humans based on a periodic low-frequency amplitude modulation of the complex pattern of high-frequency vibrations generated when sliding the finger against the texture. Periodic modulation of the envelope of these vibrations may be expected to elicit a periodic modulation of the activity of neurons^25^,^26^ responding to these vibrations, leading to the appearance of a measurable SS-EP in the EEG frequency spectrum^21^,^27^,^28^, tagging the cortical activity involved in human texture perception.

A high precision robotic device was used to slide two different sets of textures against the index finger pad at a constant horizontal speed (Fig. 1). While scanning the finger, a slight 3 Hz vertical sinusoidal movement was added to the movement such as to periodically modulate the normal force applied by the texture against the skin. This also led to a periodic modulation of the mean tangential force between the fingertip and texture. Most importantly, the sinusoidal movement induced a periodic modulation of the strength of the fine vibrations generated by the fingertip-texture interactions. Two sets of two natural textures having different spatial characteristics were tested in separate experiments: denim and baking paper in the first experiment, silk and wood in the second experiment. By concurrently recording the amplitude of the periodic variations of normal and tangential force using force sensors implemented in the texture holder, as well as the periodic modulation of high-frequency vibrations generated in the finger using an accelerometer glued on the fingertip, we were able to assess the respective contribution of these different stimulus features to the EEG responses elicited by the textures.

## Results

### SS-EPs elicited at the frequencies of amplitude modulation

Sliding the finger against all four textures elicited a significant increase of EEG signal amplitude at 3 Hz, corresponding to the frequency of the sinusoidal vertical movement of the plate (Fig. 2). The scalp topography of the elicited SS-EP was maximal over the parietal region contralateral to the stimulated fingertip. In the first experiment, the one sample t-test showed that the amplitude at 3 Hz was significantly greater than zero (denim: t(11) = 2.33, p = 0.040, 95% CI [0.003, 0.132], baking paper: t(11) = 3.65, p = 0.004, 95% CI [0.056, 0.227]). In the second experiment, the increase of amplitude at 3 Hz was also significant (silk: t(10) = 4.20, p = 0.002, 95% CI [0.031, 0.104], wood: t(10) = 4.04, p = 0.002, 95% CI [0.014, 0.050]).

A significant increase of EEG signal amplitude was also observed at the frequency of the second harmonic (6 Hz). In the first experiment, this increase was significant when the fingertip was scanned against baking paper (t(11) = 3.91, p = 0.002, 95% CI [0.035, 0.127]), but not when it was scanned against denim (t(11) = 1.42, p = 0.182, 95% CI [−0.013, 0.061]), (Fig. 2). In the second experiment, this response was significant for both textures (silk: t(10) = 4.72, p = 0.001, 95% CI [0.017, 0.048], wood: t(10) = 2.63, p = 0.025, 95% CI [0.003, 0.044]). The scalp topographies of the 6 Hz SS-EPs were similar to the scalp topographies of the SS-EPs observed at 3 Hz.

In the first experiment, the repeated-measures ANOVA used to compare the magnitude of the SS-EPs elicited by each texture at 3 Hz and 6 Hz revealed a main effect of ‘texture’ (denim vs. baking paper; F = 15.48, η^2^ = 0.585, p = 0.002). The magnitude of the SS-EP elicited by scanning the baking paper texture was greater than the magnitude of the SS-EP elicited by scanning the denim texture. There was also a main effect of ‘harmonic’ (3 Hz vs. 6 Hz; F = 6.19, η^2^ = 0.360, p = 0.030). The magnitude of the SS-EP elicited at 3 Hz was greater than the magnitude of the SS-EP elicited at 6 Hz. There was no significant interaction between the two factors (F = 0.19, η^2^ = 0.017, p = 0.671). In the second experiment, the repeated-measures ANOVA comparing the magnitude of the SS-EPs elicited by the two textures at 3 Hz and 6 Hz showed a significant main effect of ‘harmonic’ (3 Hz vs. 6 Hz; F = 4.81, η^2^ = 0.325, p = 0.053). There was no significant main effect of ‘texture’ (silk vs. wood; F = 2.39, η^2^ = 0.193, p = 0.153). There was, however a significant interaction between the two factors (F = 6.14, η^2^ = 0.380, p = 0.033). Post-hoc comparisons using paired-sample t-tests showed that the magnitude of the 3 Hz SS-EP elicited by sliding the finger against silk was significantly different compared to sliding of the finger against wood (paired samples t-test: silk vs. wood: t(10) = 2.91, p = 0.016, 95% CI [0.008, 0.063]). The magnitude of the 6 Hz SS-EP was not found significantly different for both textures (paired samples t-test: silk vs. wood: t(10) = 0.82, p = 0.430, 95% CI [−0.014, 0.032]).

### Periodic variation of the normal force between the texture and the index fingertip

As expected, the 3 Hz sinusoidal vertical movement of the plate against the fingertip induced a periodic variation of the normal force (Experiment 1: baking paper: t(11) = 11.06, p < 0.001, 95% CI [0.156, 0.234], denim: t(11) = 9.93, p < 0.001, 95% CI [0.137, 0.216]; Experiment 2: silk: t(10) = 8.73, p < 0.001, 95% CI [0.121, 0.205], wood: t(10) = 14.54, p < 0.001, 95% CI [0.165, 0.225]) (Fig. 3). In contrast, no significant variation of normal force was observed at 6 Hz for either of the four textures (baking paper: t(11) = 0.36, p = 0.725, 95% CI [−0.012, 0.017], denim: t(11) = 0.84, p = 0.422, 95% CI [−0,005, 0.011], silk: t(10) = 1.44, p = 0.180, 95% CI [−0.009, 0.004], wood: t(10) = 1.38, p = 0.197, 95% CI [−0.002, 0.001]).

Contrasting with the results of the EEG analysis showing that sliding the finger against baking paper elicited SS-EPs of significantly greater magnitude as compared to denim (Experiment 1), and that sliding the finger against silk elicited SS-EPs of greater magnitude as compared to wood (Experiment 2), the periodic 3 Hz oscillations of normal force obtained for each of the two sets of textures were not significantly different (paired samples t-test: denim vs. baking paper: t(11) = 1.81, p = 0.101, 95% CI [−0.004, 0.041], silk vs. wood: t(10) = −1.50, p = 0.165, 95% CI [−0.078, 0.015]) (Fig. 3).

### Periodic variation of the tangential force between the texture and the index fingertip

A significant 3 Hz sinusoidal variation of the tangential force was observed for all four textures (Experiment 1: baking paper: t(11) = 13.67, p < 0.001, 95% CI [0.034, 0.047], denim: t(11) = 11.50, p < 0.001, 95% CI [0.039, 0.058]; Experiment 2: silk: t(10) = 7.50, p < 0.001, 95% CI [0.026, 0.049], wood: t(10) = 17.11, p < 0.001, 95% CI [0.043, 0.055]) (Fig. 3). In contrast, there was no significant variation of the tangential force at 6 Hz (baking paper: t(11) = 1.00, p = 0.342, 95% CI [−0.001, 0.001], denim: t(11) = −1.45, p = 0.179, 95% CI [−0.001, 0.001], silk: t(10) = 1.84, p = 0.095, 95% CI [−0.001, 0.001], wood: t(10) = 1.15, p = 0.279, 95% CI [−0.001, 0.002]).

In the first experiment, the magnitude of the 3 Hz oscillation of tangential force was smaller for baking paper as compared to denim (t(11) = −2.58, p = 0.027, 95% CI [−0.142, −0.001]). This contrasted the finding that the magnitude of the SS-EPs elicited by sliding the finger against baking paper was significantly greater than the magnitude of the SS-EPs elicited by sliding the finger against denim. Similarly, in the second experiment, the magnitude of the 3 Hz oscillation of tangential force tended to be smaller for silk as compared to wood (silk vs. wood: t(10) = −2.07, p = 0.065, 95% CI [−0.023, 0.001]), whereas the SS-EP elicited by the silk texture was significantly greater than the SS-EP elicited by the wood texture.

### Periodic modulation of fine fingertip vibrations

A 52–300 Hz band-pass filter was applied to the vertical acceleration signals recorded by the accelerometer glued on the finger, such as to obtain a measure of the high-frequency vibrations generated in the index fingertip. A Hilbert transform was then used to estimate the time course of the envelope of these high-frequency vibrations. Frequency analysis of the envelopes showed that, for both sets of textures, the sinusoidal sliding movement generated a significant 3 Hz modulation of the high-frequency vibrations induced by the interactions between the texture and the index fingertip (Experiment 1: denim: t(8) = 5.79, p < 0.001, 95% CI [105.8, 246], baking paper: t(8) = 7.64, p < 0.001, 95% CI [182.01, 339.4]; Experiment 2: silk: t(10) = 7.05, p < 0.001, 95% CI [220.8, 425.1], wood: t(10) = 3.01, p = 0.013, 95% CI [37.77, 254.3]). No significant signal was observed at 6 Hz for any of the textures (baking paper: t(8) = −0.56, p = 0.593, 95% CI [−31.73, 13.39], denim: t(8) = 0.09, p = 0.933, 95% CI [−18.61, 20.06], silk: t(10) = 0.01, p = 0.990, 95% CI [−35.42, 35.84], wood: t(10) = 0.30, p = 0.767, 95% CI [−9.676, 12.73]) (Fig. 3).

In line with the results of the EEG analysis showing that sliding the finger against baking paper elicited a significantly greater SS-EP as compared to denim (experiment 1), the magnitude of the 3 Hz modulation of high-frequency vibrations induced by sliding the fingertip against baking paper was significantly greater as compared to denim (t(8) = 2.715, p = 0.026, 95% CI [12.76, 156.8]). Similarly, in the second experiment, the texture that induced the strongest SS-EP (silk as compared to wood) was also the texture generating the strongest 3 Hz modulation of high-frequency vibrations (t(10) = 3.596, p = 0.005, 95% CI [67.29, 286.6]).

### Amplitude modulation of high-frequency EEG oscillations

The same band-pass filter (52–300 Hz) and Hilbert transform was applied to the recorded EEG signals, such as to obtain an estimate of the envelope of the high-frequency content of the EEG signals. Frequency analysis of these signals did not reveal any significant amplitude modulation of high-frequency EEG oscillations, both at 3 Hz and at 6 Hz. The one sample t-test showed that the amplitude of the envelope at 3 Hz was not significantly different from zero (Experiment 1: denim: t(11) = −0.56, p = 0.588, 95% CI [−0.002, 0.001], baking paper: t(11) = −0.06, p = 0.951, 95% CI [−0.004, 0.004]; Experiment 2: silk: t(10) = 0.90, p = 0.392, 95% CI [−0.001, 0.001], wood: t(10) = 0.58, p = 0.576, 95% CI [−0.003, 0.005]). Likewise, the amplitude at 6 Hz was not significantly different from zero for all four textures (Experiment 1: denim: t(11) = −0.42, p = 0.686, 95% CI [−0.003, 0.002], baking paper: t(11) = 0.63, p = 0.539, 95% CI [−0.003, 0.006]; Experiment 2: silk: t(10) = 1.87, p = 0.091, 95% CI [−0.001, 0.002], wood: t(10) = 1.58, p = 0.146, 95% CI [−0.001, 0.003]).

## Discussion

The aim of this study was to develop a non-invasive method to characterise, in humans, the cortical activity elicited by sliding the fingertip against the surface of a natural texture. For this purpose, a slight periodic vertical movement of the texture against the fingertip was added to the horizontal sliding movement, such as to generate a periodic modulation of the fine mechanical vibrations generated by the texture-fingertip interactions. This periodic modulation was expected to elicit a steady-state evoked potential (SS-EP) in the recorded scalp EEG signals, appearing at the frequency of the sinusoidal movement, and tagging the neural activity related to the processing of texture-induced vibrations.

Sliding the finger against two sets of textures (baking paper vs. denim and silk vs. wood) elicited a significant SS-EP at the frequency of the sinusoidal movement (3 Hz) for all textures. Sliding the finger against three of the four textures (i.e. baking paper, silk and wood) elicited additional SS-EPs at 6 Hz, corresponding to the frequency of the second harmonic. The scalp topographies of the elicited brain responses were maximal over the parietal regions contralateral to the stimulated hand, suggesting that they originated, at least in part, from the contralateral SI.

These brain responses could be related to (1) the periodic 3 Hz modulation of the high-frequency vibrations generated by sliding the texture against the fingertip. However, they could also be related to (2) the periodic 3 Hz oscillation of the normal force applied against the fingertip and/or (3) the periodic 3 Hz oscillation of the tangential force applied against the fingertip. Indeed, each of these three aspects of the mechanical fingertip stimulation can be expected to activate peripheral mechanoreceptors in a periodic fashion and, hence, contribute to the observed SS-EPs.

Interestingly, the magnitude of the SS-EPs elicited by sliding the fingertip against the baking paper texture was significantly greater than the magnitude of the SS-EPs elicited by sliding the fingertip against the denim texture. A significant difference was also found when comparing the magnitude of the SS-EPs elicited by sliding the fingertip against silk as compared to wood. This differed from the difference in magnitude of the periodic variations of both the normal force and the tangential force. Indeed, the periodic variation of the normal force generated when sliding the fingertip against the pairs of textures was not significantly different. Furthermore, in both experiments, the textures eliciting the strongest SS-EPs were the textures generating the smallest variations in tangential force (baking paper as compared to denim, silk as compared to wood).

In contrast and, most importantly, in line with the EEG results, the magnitude of the amplitude modulation of the high-frequency vibrations generated in the index fingertip was significantly greater when sliding the fingertip against baking paper as compared to denim, and for silk as compared to wood. This suggests that a substantial part of the recorded SS-EPs was actually related to the cortical processing of the fine vibrations conveying texture information.

Previous studies have shown that scalp EEG is also able to sample high-frequency neural activities, such as high-frequency brainstem activity elicited by auditory stimulation^29^,^30^ or the transient burst of high-frequency oscillations (up to 600 Hz^31^) which can be elicited by transcutaneous electrical stimulation of the median nerve and is thought to originate, at least in part, from activity generated in SI^32^. Therefore, by applying a high-pass filter and a Hilbert transform to the recorded EEG signals, we examined whether sliding the finger against the two sets of textures elicited a periodic modulation of high-frequency EEG activities. However, this approach did not allow us to identify any significant responses. Given that only a small fraction of SI neurons may be able to follow such high frequencies^18^, and considering that the scalp acts as a low pass filter attenuating high-frequency signals^33^, the magnitude of the EEG signal generated by amplitude modulation of high-frequency activity within these neurons was probably insufficient to allow their reliable identification. Future studies using, for example, subdural electrode grids implanted for the presurgical evaluation of medically intractable partial epilepsy might make it possible to identify high-frequency activities generated by the tactile exploration of textures in humans.

Our results agree with the results of EEG studies showing that amplitude modulation of a pure sinusoidal mechanical vibration (e.g. a 128 Hz vibration modulated at frequencies ranging between 5–40 Hz^34^) elicits a significant SS-EP at the frequency of amplitude modulation, with a topographical distribution compatible with activity predominantly originating from SI contralateral to the site of stimulation^21^,^23^,^27^,^28^. Such as the SS-EPs recorded in the present study, SS-EPs elicited by amplitude modulation of a higher-frequency vibration can be explained by the fact that the mean firing rate of neurons responding to the vibro-tactile input, and the total number of activated afferents are both dependent on vibration magnitude^35^. Indeed, both factors can be expected to strongly determine, within SI, stimulus-evoked summated post-synaptic activity, i.e. the main contributor of scalp EEG signals.

To summarise, this study presents a novel method to explore the cortical processing of sensory information generated by the dynamic tactile exploration of natural textures in humans. This creates new opportunities to study dynamic touch. For instance, it could be used to better understand the differences between “active” and “passive” touch, i.e. dynamic touch respectively with and without voluntary movement^36^,^37^.

## Methods

The ethics committee on human research of Université catholique de Louvain approved the study (Virtual Prototyping of Tactile Displays, PROTOTOUCH-317100). All participants gave written informed consent. The investigation conformed to the principles of the Declaration of Helsinki.

### Participants

Twelve healthy right-handed volunteers participated in the first experiment (aged 19 to 38 years, 7 females) and eleven right-handed volunteers participated in the second experiment (aged 25 to 35 years, 6 females). The subjects were seated comfortably in front of the apparatus.

### Dynamic tactile stimulation

**Figure 1:**
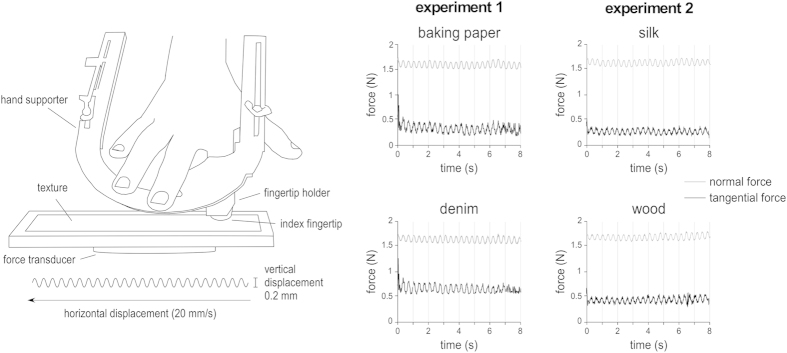
Experimental setup. A robot was used to scan the right index fingertip against a natural texture at a constant 20 mm/s velocity, and a constant mean normal force of 1.5 N. To periodically modulate the amplitude of the high-frequency vibrations elicited by the fingertip-texture interaction, a 3 Hz 0.2 mm vertical sinusoidal displacement was added to the horizontal scanning movement. Normal and tangential forces applied against the finger were measured using a forces and torque transducer. The right graphs show, in one illustrative trial, the periodic modulation of the normal force and tangential force generated by the sinusoidal movement against each texture (x-axis: time, in seconds; y-axis: force, in Newton). Each displacement lasted 8.2 s. The displayed time courses correspond to the eight seconds of periodic displacement used for the EEG analysis.

**Figure 2:**
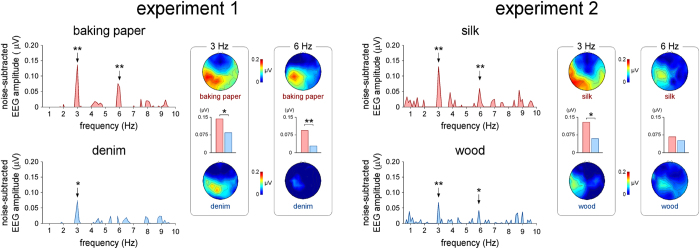
Group-level average frequency spectrum of the EEG signals recorded while the right index fingertip was scanned against each set of textures in the two experiments (baking paper vs. denim, silk vs. wood). X-axis: frequency (Hz). Y-axis: noise-subtracted amplitude averaged across all left parietal electrodes. Significant increases in amplitude at expected SS-EP frequencies (3 Hz and 6 Hz) are shown by the vertical arrows (*p < 0.05; **p < 0.005; t-test against zero). The scalp topographies show the topographical distribution of the signals measured at 3 Hz and 6 Hz. Note the greater response elicited when scanning the baking paper texture as compared to the denim texture, and the silk texture as compared to the wood texture. Also note that the elicited SS-EPs are maximal over the parietal region contralateral to the stimulated index finger.

**Figure 3:**
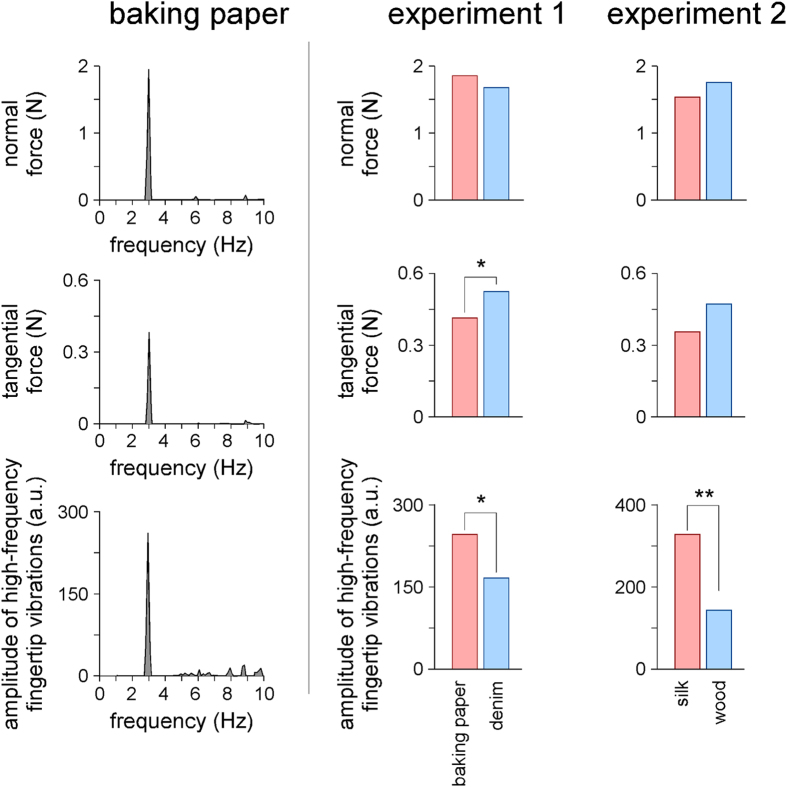
Variations in normal and tangential forces, and variations in the amplitude of high-frequency vibrations recorded by the accelerometer while sliding the index fingertip against the four textures. Average frequency spectra representing these variations are illustrated for one texture (baking paper). The bar graphs show the amplitude of the 3 Hz variations for each of the four textures. Note that the 3 Hz 0.2 mm vertical sinusoidal movement added to the sliding motion generated a periodic 3 Hz variation of normal and tangential forces. Also note that this sinusoidal movement generated a 3 Hz modulation of the amplitude of high-frequency vibrations (52–300 Hz) in the fingertip. Such as the SS-EPs shown in Fig. 2, the magnitude of the amplitude modulation of high frequency vibrations was greater for baking paper vs. denim, and for silk vs. wood. In contrast, the modulation of normal force was similar for the two textures in both experiments, and the modulation of tangential force was smaller for baking paper vs. denim and for silk vs. wood.

A schematic view of the experimental setup is shown in Fig. 1. Two different sets of textures (Experiment 1: denim vs. baking paper, Experiment 2: silk vs. wood) were glued onto two flat glass plates (length: 20 cm, width: 3 cm). To avoid any changes of the textures due to repeated sliding against the fingertips, the textures were renewed for each participant.

A forces and torque transducer (Mini40 F/T transducer, ATI Industrial Automation, USA) was mounted on a four-axis robot (four-axis SCARA HS series 4535G. DENSO, USA), driven by the LabVIEW (National Instruments, USA) software. The forces and torque transducer provided online measures of contact forces (normal and tangential forces to the surface), which were sampled at 1000 Hz using an analogue-to-digital data acquisition system (DAQ 6071E, National Instruments, Austin, TX, USA). The index of the participant was maintained using a custom hand support. The texture plates were mounted on the force sensor, and displaced from left to right at a constant 20 mm/s horizontal velocity during 8.2 seconds. The vertical position of the plate was adjusted such that the average normal force was 1.5 N. The choice of this force and scanning velocity was justified by the fact that, in a preliminary pilot experiment, participants reported a comfortable and natural feeling of tactile exploration for the whole duration of the sliding movement. During the scanning, an additional 3 Hz sinusoidal vertical displacement was added to the movement, with a peak-to-peak amplitude of 0.2 mm. Contact between the plate and finger was maintained during the entire scanning. This sinusoidal movement of the plate against the fingertip led to a periodic variation of the average normal force and tangential force (Fig. 1). Most importantly, it also led to a periodic amplitude modulation of texture-induced vibrations in the exploring finger. The same initial position on the plate (along the x-axis) was achieved for all participants.

### EEG data acquisition

The EEG was recorded using 64 Ag–AgCl electrodes placed on the scalp according to the International 10/10 system (Waveguard64 cap, Cephalon A/S, Denmark), against an average reference. Ocular movements and eye blinks were recorded using an additional bipolar channel and two surface electrodes placed at the upper-left and the lower-right rides of the right eye. Electrode impedances were kept below 10 kΩ. Signals were amplified and digitised using a sampling rate of 1000 Hz (64-channel high-speed amplifier, Advanced Neuro Technology, The Netherlands). During the recording, participants were instructed to attend a fixation cross. Furthermore, they listened to white noise presented through headphones to avoid any contribution of auditory input to the recorded EEG responses.

Each experiment consisted of a total of four blocks: two blocks of 20 strokes against the fingertip using one texture and two blocks of 20 strokes using the second texture. The order of the blocks was counterbalanced across participants. At the beginning of each stroke, a trigger was sent to the EEG recording system, marking the trial onset. Each stroke lasted 8.2 seconds. A variable pause of 4–6 s was introduced between the offset of each stroke and the onset of the following stroke.

### Accelerometer data acquisition

For the first experiment, nine (out of twelve) healthy volunteers took part in a control experiment, performed at the end of the main experiment. In the second experiment, all eleven volunteers took part. The aim of this control experiment was to estimate the frequency spectrum of the vibrations induced by scanning the index fingertip against the four textures. A lightweight 3-axis accelerometer (Analog Devices, Pololu, ADXL 335) was strapped around the right index fingertip. The three analogue signals of the accelerometer were sampled at 1000 Hz using three auxiliary channels of the EEG amplifier. A total of ten trials were recorded for each type of texture.

### EEG data analysis

The EEG data were analysed in MATLAB (The Mathworks, MA) using the Letswave toolbox (http:/ www.nocions.org/letswave; see also^38^). The data were imported and high-pass filtered at 0.1 Hz to remove slow drifts in the recorded signals. EEG epochs were segmented from +0.2 s to +8.2 s relative to the onset of each trial. These EEG segments corresponded to the time interval during which the finger moved passively against the plate at a constant velocity. The first 0.2 s were discarded from the analysis because of the large amplitude broadband vibrations occurring at movement onset.

Artifacts due to eye blinks or eye movements were removed using a validated method based on an independent component analysis (FastICA algorithm^39^). Finally, the signals were re-referenced against the frontal electrode Fz.

For each subject and for each texture type, EEG epochs were averaged across trials to enhance the signal-to-noise ratio by attenuating the contribution of activities that were not phase-locked across trials. The obtained average waveforms were then transformed in the frequency domain using a discrete Fourier Transform^40^, yielding a frequency spectrum ranging from 0 to 500 Hz with a frequency resolution of 0.125 Hz^41^. Amplitude spectra were then obtained using the modulus of the complex Fourier coefficients.

Assuming additive noise, the obtained EEG frequency spectra may be expected to correspond to the sum of (1) EEG activity induced by the tactile stimulation and (2) remaining unrelated background noise due, for example, to spontaneous EEG activity, muscle activity or eye movements. Thus, to obtain estimates of the magnitude of the elicited SS-EPs, the contribution of this noise was removed by subtracting, at each bin of the frequency spectra, the average amplitude measured at surrounding frequencies (±0.3–0.5 Hz relative to the expected SS-EP frequency). This procedure is justified by the fact that, in the absence of an SS-EP, the amplitude at a given frequency can be expected to be similar to the amplitude of the mean of the surrounding frequencies^42^,^43^. Hence, in the absence of an SS-EP, the noise-subtracted amplitude should tend towards zero.

#### SS-EPs elicited at the frequency of amplitude modulation

We expected that amplitude modulation of the vibrations induced by scanning the texture against the fingertip would elicit an SS-EP at the frequency of modulation (3 Hz) and, possibly, its harmonics. The amplitude of the noise-subtracted EEG spectra at 3 Hz and 6 Hz were used as measures of these SS-EPs.

#### Amplitude modulation of high-frequency EEG oscillations

In addition, we hypothesised that if the complex high-frequency spectral pattern of the vibrations induced by scanning the texture against the fingertip elicited activity within neuronal populations frequency-locked to these vibrations^18^, 3 Hz amplitude modulation of these mechanical vibrations could lead to a 3 Hz amplitude modulation of high-frequency EEG oscillations. To investigate whether such high-frequency activities were present in our EEG recordings, single-trial EEG epochs were filtered using a 52–300 Hz bandpass Butterworth filter, thereby retaining only high-frequency EEG activities. A Hilbert transform was then used to estimate the envelope of these high-frequency oscillations as a function of time. Finally, the signals were averaged in the time domain and an FFT was applied to the data. After applying the noise-subtraction procedure, the amplitude of the signals at 3 Hz and 6 Hz was estimated.

### Normal force, tangential force and accelerometer data analysis

To assess the periodic changes in normal force and tangential force induced by the sinusoidal sliding movement of the plate against the index fingertip, as well as the periodic modulation of the frequency content of the high-frequency vibrations induced by scanning the textured surfaces against the fingertip, the measures of normal and tangential force obtained from the forces and torque transducer, as well as the measures of vertical accelerations obtained from the accelerometer were analysed in the frequency domain, using the same procedures used to analyse the EEG data.

### Statistical Analyses

Statistical analyses were performed using SPSS 22 (IBM, USA). Significance level was set at p < 0.05.

To assess the significance of the elicited SS-EPs, the noise-subtracted spectra of the EEG signals measured at parietal and temporal electrodes contralateral to the stimulated fingertip were averaged (i.e. T7, C1, C3, C5, CP1, CP3, CP5, P1, P3, P5, P7, TP7). Considering their position relative to SI, and considering that the signals were referenced to the midline frontal electrode Fz, these electrodes may be expected to best capture activity originating from SI^44^. One-sample t-tests against zero were then used to assess whether the noise-subtracted amplitudes measured at the expected SS-EP frequencies (3 Hz and 6 Hz, corresponding to the second harmonic) were significantly greater than zero.

To compare the SS-EPs elicited by each of the two sets of textures, a two-way repeated measures ANOVA was then performed with the factors ‘texture’ (Experiment 1: denim vs. baking paper, Experiment 2: silk vs. wood) and ‘harmonic’ (first vs. second harmonic). The assumption of sphericity was tested using Mauchly’s test. In those cases where the data violated the assumption of sphericity, F-values were corrected using the Greenhouse-Geisser procedure. When appropriate, post-hoc comparisons were performed using paired-sample t-tests.

## Additional Information

**How to cite this article**: Moungou, A. *et al.* EEG frequency tagging to explore the cortical activity related to the tactile exploration of natural textures. *Sci. Rep.* **6**, 20738; doi: 10.1038/srep20738 (2016).
